# Extended abdominoperineal resection in women: the barbadian experience

**DOI:** 10.1186/1477-7800-4-1

**Published:** 2007-01-10

**Authors:** Andrew P Zbar, Radhakanth K Shenoy, Antonio Chiappa

**Affiliations:** 1 Professorial department of surgery the university of thewest indies, Queen Elizabeth Hospital, Barbados; 2 Department of Radiotherapy, Queen Elizabeth Hospital, Barbados; 3 Department of General Surgery, European Institute of Oncology, Milano, Italy

## Abstract

**Background and objectives:**

We report our results of a selective approach to primary direct appositional vaginal repair versus transverse rectus abdominis flap repair (TRAM) in patients with extensive rectal/anal cancer or in cases with primary cancer of cervix, vagina or vulva involving the anal canal and anal sphincters.

**Methods:**

Eighteen female patients (mean age: 62.9 years; range: 44–81 years) with a median follow-up of 14 months (range: 2–36 months) undergoing extended abdominoperineal reconstruction **with total mesorectal excision **between May 2002 and September 2005, were studied.

**Results:**

Twelve patients underwent an extended abdominoperineal resection with hysterectomy and vaginectomy, with 6 patients undergoing primary TRAM flap reconstruction **following pelvic exenteration. Exenterative procedures were performed in 2 cases of primary vaginal cancer, following Wertheim hysterectomy for carcinoma of the cervix with recurrence after radiation and in 2 further cases of anal cancer with extensive pelvic recurrence after primary chemoradiation**. Fifteen cases are alive on follow-up with no evidence of disease; 2 patients who had recurrent carcinoma of the cervix and who underwent TRAM flap reconstruction, **have **recurrent disease after 5 and 6 months of follow-up, respectively.

**Discussion:**

Our experience shows that careful primary closure of an extended abdominoperineal resection wound is effective and safe. Our **one **case of wound breakdown after primary repair underwent external beam and intracavitary irradiation **primarily with wound breakdown of a primary repair followed by a delayed pedicled graciloplasty**. TRAM flap reconstruction has been reserved in our unit for patients undergoing total pelvic extenteration. In general, we would recommend the use of TRAM flap reconstruction in younger sexually active patients where there has been external irradiation combined with brachytherapy.

## Introduction

Extended abdominoperineal resection, (radical excision of the rectum and perineum with partial or near total vaginectomy), may be required in extensive rectal cancer treated initially with neoadjuvant chemoradiation, in some patients with squamous carcinoma of the anus and anal canal where there is limited response or recurrence **and **in **some **patients with primary carcinoma of the cervix, vagina or vulva where there is rectal and/or anal involvement [1-3]. Most if not all of these patients have already undergone pelvic and perineal irradiation sometimes with **additional **brachytherapy, where there is requisite need for radical excision in an irradiated field and concomitant perineal and/or vaginal reconstruction.

Perineal wounds in such patients are at considerable risk for dehiscence and delayed healing when primarily repaired, [4] sometimes requiring specialist secondary distant fascio- or myocutaneous flap repair [5]. It has been argued that this is best prevented by the initial utilization of unirradiated tissue for composite reconstruction of the perineum with the routine creation of a neovagina [6], although others have suggested that this approach should be selective particularly being reserved for complex vaginal defects in patients who are sexually active where a neovagina is required [7]. **This approach may also be used successfully **in patients who have undergone combined external radiation and brachytherapy or where repeat irradiation for recurrent carcinoma has been utilized [8]. The commonest myocutaneous flap recommended in such cases is the vertical or transverse rectus abdominis (TRAM) flap as originally described by Taylor and colleagues [9] which provides a large neurovascularized skin paddle with a reliable muscle pad which can be rotated through a wide arc and where the donor defect can be primarily closed without mesh insertion [10]. The simpler alternative if adequate resection margins can be achieved is extended abdominoperineal resection with partial or near total vaginal excision and high posterior vaginal and fourchette reconstruction, [11] where the risk of breakdown with primary repair is balanced against the procedural complexity. We report our results over the last 3 years in Barbados of a selective approach to primary direct appositional vaginal repair versus TRAM flap repair in these specialized cases as performed by a single colorectal surgeon (APZ).

## Patients and methods

**Figure 1:**
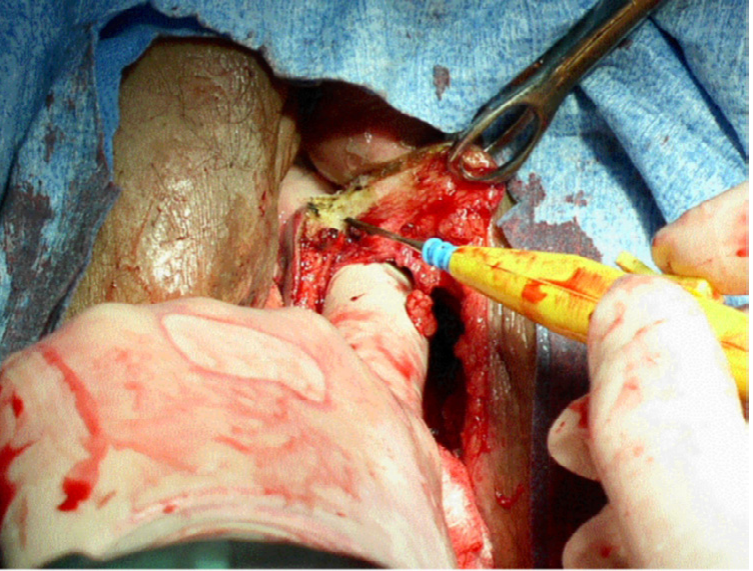
The beginnings of the vaginectomy *en bloc *with the perineal rectal resection.

**Figure 2:**
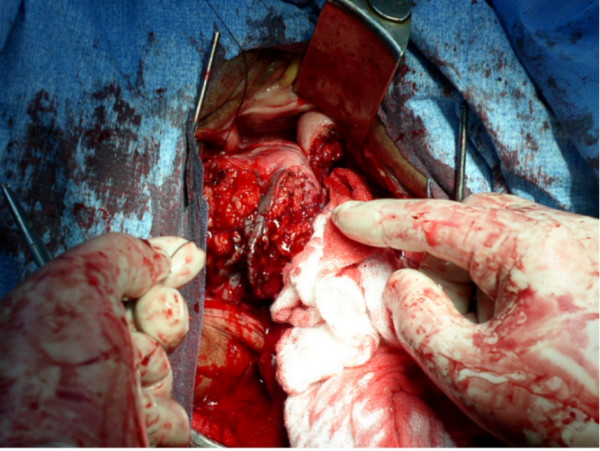
Ligation of the inferior vaginal venous plexus.

Simple extension of the abdominoperineal resection with high posterior vaginectomy [12] has been well described and is shown in Figure 1, 2, 3. Formal vaginal and perineal reconstruction is performed by aligning the new fourchette after removing the entire posterior vaginal wall where necessary and by linking this to the apex of the perineal wound. High posterior vaginectomy requires ligation of the inferior vaginal venous plexi on both sides with care being taken at the posterior vaginal apex not to injure the lower end of the ureter on either side. Our group has described the use of the TRAM flap in the past for total pelvic exenteration [3] with double ostomy (ileal conduit and colostomy), however, the skin paddle may also be used for the construction of a neovagina as described by Bell and colleagues [6] as well as by others [13]. The TRAM flap for total pelvic exenteration is shown in Figure 4, 5, 6. Preoperative assessment of patients included endorectal ultrasonography, CT scanning and MR imaging where appropriate. The latter modality was used in recurrent cases to assess presacral infiltration in the sagittal plane although there were no cases where sacrectomy was required. One patient with a recurrent mass in the rectovaginal septum with carcinoma of the anus who received primary chemoradiation underwent transperineal sonography (Figure 7) which demonstrated the septal mass, the complete excision of which was guided by intraoperative ultrasound. The transcutaneous ultrasound technique has been described before by our group in organic and functional disease [14].

**Figure 3:**
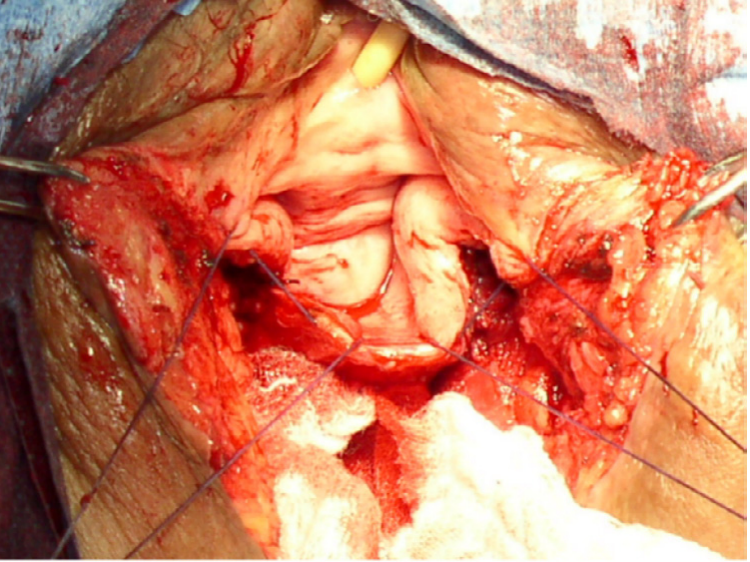
Posterior vaginal reconstruction.

**Figure 4:**
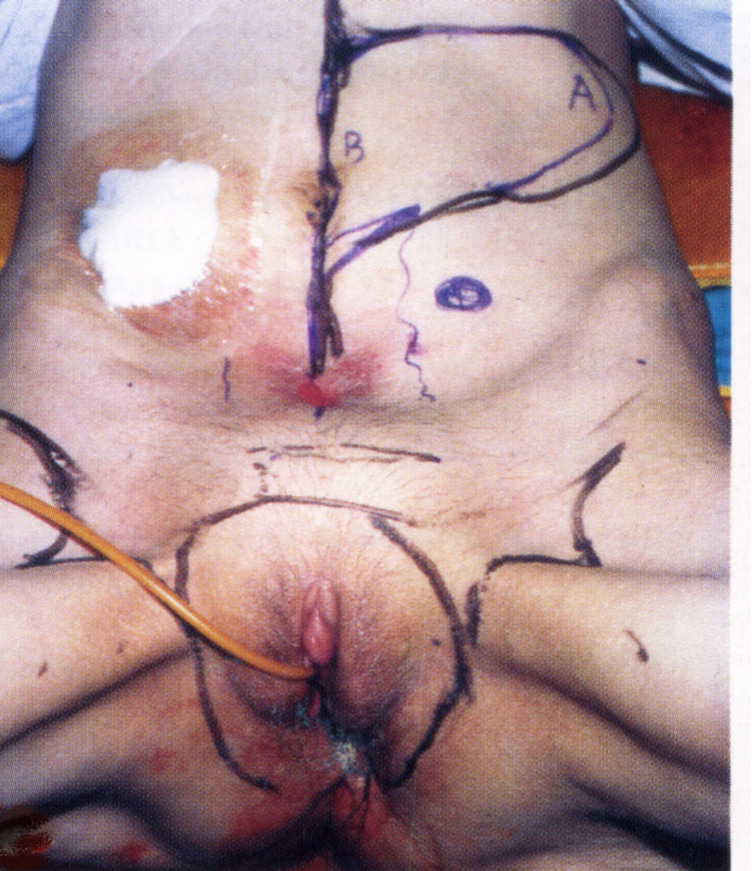
TRAM flap construction for total pelvic exenteration combined with bilateral inguinal lymphadenectomy (Flap design).

**Figure 5:**
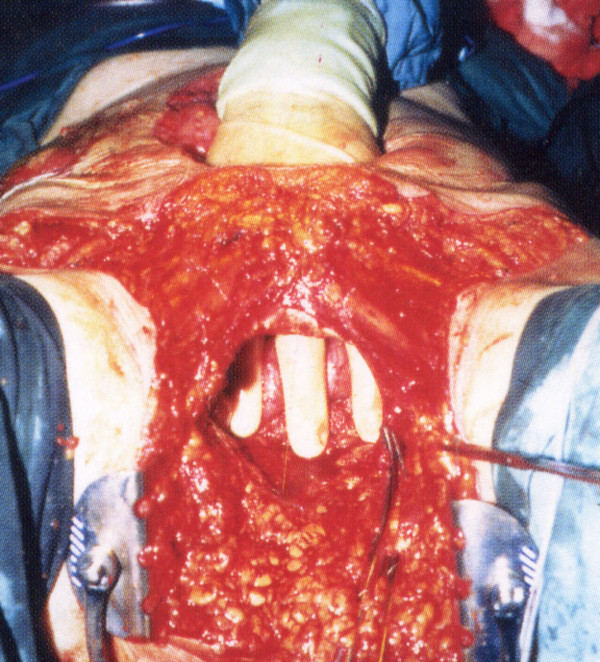
TRAM flap construction for total pelvic exenteration combined with bilateral inguinal lymphadenectomy (Exenteration defect).

**Figure 6:**
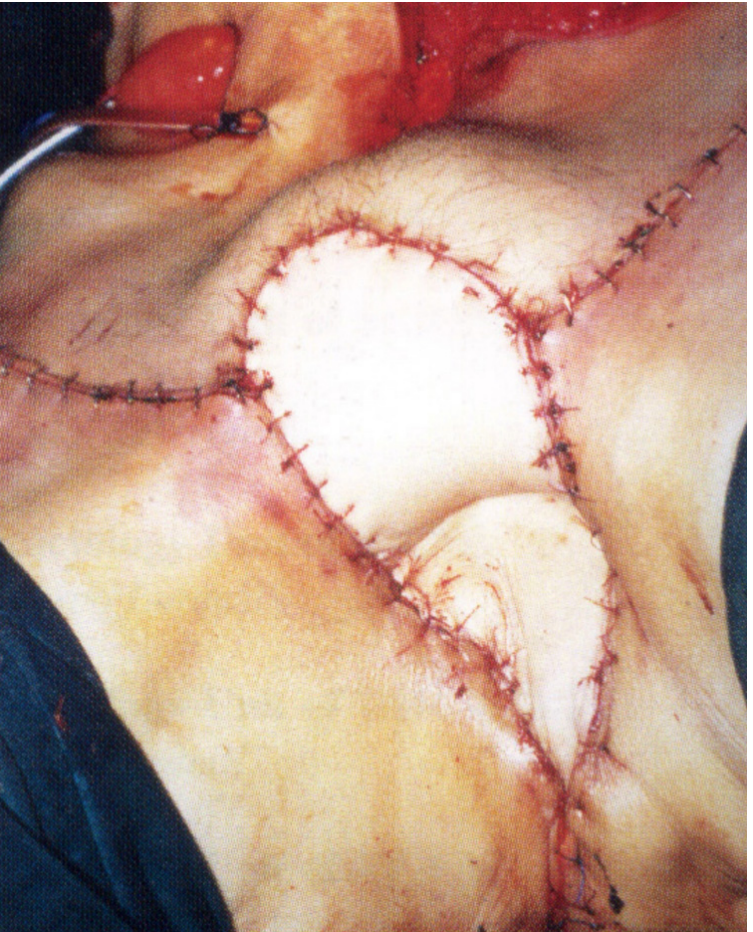
TRAM flap construction for total pelvic exenteration combined with bilateral inguinal lymphadenectomy (Appearance of the flap in position). (Reprinted with permission Springer-Verlag Publishers) from Zbar AP, Nishikawa H, Beer-Gabel M (2001) BeerGabel M. Use of the V-RAM flap in reconstruction after total pelvic exenteration for recurrent vulval cancer involving the anal sphincter. Techn Coloproctol 5: 66)

**Figure 7:**
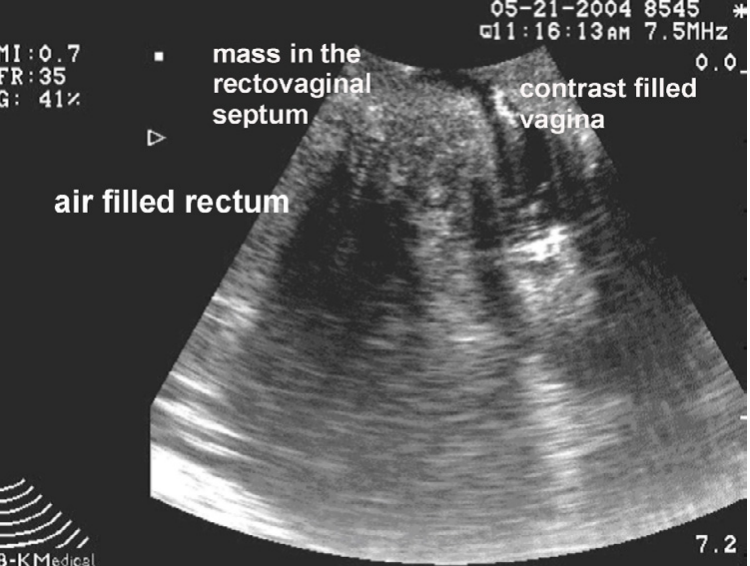
Sagittal transperineal sonography showing the recurrent mass in the same patient occupying the rectovaginal septum. The vagina has been filled with acoustic contrast.

## Results

Table 1 shows the clinical characteristics of 18 patients undergoing extended abdominoperineal reconstruction (mean age 62.9 years, range 44–81 years) between May 2002 and September 2005. The median follow-up was 14 months (range 2–36 months). Twelve patients underwent an extended abdominoperineal resection with hysterectomy and vaginectomy (2 anal cancers, 8 rectal cancers and 2 cervical carcinomas) with 6 TRAM flaps as part of total pelvic exenterations **primarily **(2 **for **carcinomas of the vagina, 2 anal **recurrent **carcinomas **after primary chemoradiation **and 2 recurrent cervical carcinomas **after Wertheim hysterectomies and high-dose radiotherapy**). All patients with rectal and anal carcinoma received preoperative chemoradiation with a median radiation dose of 50 cGy with anal cancer patients routinely receiving elective bilateral inguinal irradiation. Patients with cervical carcinoma also received intracavitary radiation (21 cGy in 6–13 fractions delivered to point A). The chemotherapy used in each case of rectal cancer was 5-Fluorouracil (425 mg/m^2^) plus Leukovorin rescue (20 mg/m^2^) with the anal cancers undergoing a modified Nigro régime utilizing 5-Fluorouracil alone [15]. Histological assessment of all cases showed only one patient with circumferential margin involvement in the perineal aspect of the specimen in a patient receiving neoadjuvant chemoradiation for an advanced rectal cancer. This patient died 9 months later from a cerebral metastasis with no evidence of local recurrence. Eight patients had involved lymph nodes with 2 of these patients with anal cancer presenting initially with involved inguinal lymph nodes. Fifteen patients are alive with no evidence of disease on follow-up with both patients who had recurrent carcinoma of the cervix and who underwent total pelvic exenteration and TRAM flap construction **who have **recurrent disease after 5 and 6 months of follow-up respectively (Table 1).

**Table 1:** Characteristics of 18 patients treated with extended abdominoperienal resection (2002–2005)

AGE (Years)	CANCER TYPE	ICRT	INGUINAL NODES	OPERATION	FOLLOW-UP (MONTHS)	OUTCOME
56	ANUS			EAPR	36	AWOD
60	CERVIX	+		EAPR	30	AWOD
62	VAGINA			TRAM	28	AWOD
48	VAGINA			TRAM	27	AWOD
70	RECTUM			EAPR	22	AWOD
71	RECTUM			EAPR	22	AWOD
61	RECTUM			EAPR	20	AWOD
64	RECTUM			EAPR	19	AWOD
67	RECTUM			EAPR	16	AWOD
44	ANUS			TRAM	12	AWOD
50	ANUS		+	EAPR	11	AWOD
55	CERVIX	+		EAPR	9	AWOD
53	RECUR. CERVIX			TRAM	6	RECUR
78	RECUR. CERVIX			TRAM	5	RECUR
81	ANUS		+	TRAM	5	AWOD
72	RECTUM			EAPR	5	AWOD
70	RECTUM			EAPR	3	AWOD
70	RECTUM			EAPR	2	AWOD

ICRT = Patients receiving intracavitary radiation

EAPR = Extended abdominoperineal resection with primary closure

TRAM = Transverse rectus abdominis myocutaneous flap repair

AWOD = Alive without disease

RECUR = Recurrence

The median hospital stay for patients undergoing simple extended abdominoperineal resection was 17 days (range 11–23 days) and for TRAM flap was 26 days (range 19–63 days). Mesh insertion was not used for any case undergoing a TRAM flap with one patient having an incisional hernia of the donor site at one year of follow-up. The same patient had apical cutaneous flap necrosis requiring minor débridement and dressings which extended her hospital stay. One patient undergoing simple extended abdominoperineal resection for an advanced cervical carcinoma, (who received both external radiotherapy and brachytherapy), experienced breakdown of the vaginal repair necessitating a delayed graciloplasty 4 months after the initial procedure which proved successful. No patient was sexually active through the period of follow-up.

## Discussion

The outcome is reported of a select group of patients presenting to a specialist colorectal unit with extensive rectal carcinoma following neoadjuvant therapy, recurrent anal squamous cell carcinoma after primary chemoradiation and primary or recurrent cervical and vaginal carcinoma involving the anus where the surgical decision was made either for extended abdominoperineal resection with high posterior vaginectomy or for total pelvic exenteration with TRAM flap perineal reconstruction. No patients underwent neovaginal reconstruction using the TRAM flap technique. All patients received preoperative radiation with 2 cases receiving both external and intracavitary radiotherapy. Eleven out of 12 patients with extended vaginectomy showed primary healing and 5 of the 6 cases undergoing TRAM flap reconstruction healed primarily.

In this complicated setting using high dose perineopelvic radiation, delayed perineal healing may be expected with chronic perineal sinus formation at 3 months after abdominoperineal excision being reported in between 15–65% of patients [16-18]. Our results in this small series show that careful primary closure of an extended abdominoperineal resection wound is effective. **The **only case of dehiscence resulting in a patient undergoing combined external and intracavitary irradiation being successfully treated with a pedicled graciloplasty. The TRAM flap has been reserved in our patients only for those undergoing total pelvic exenteration and although its unselected use has been shown to be safe as a primary treatment, [6,10] the simpler technique of primary closure of an extended perineal resection is generally recommended in these patients. The TRAM flap affords the creation of an oblique skin paddle which exceeds the dimensions of the muscle pad and it can be selectively employed where there is an extensive perineal defect or in sexually active patients where a neovaginal reconstruction is planned [19]. **In **selected cases it may be combined with a myoperitoneal flap to reduce flap-related morbidity [20]. Alternatives may include omental flap vaginoplasty and perineoplasty, [21] or the use of a range of new fully or partially bioabsorbable composite meshes [22] which appear to provoke less adhesion formation but are associated with more infections and bowel or urinary fistulas [23].

It is accepted that there is considerable psychosexual morbidity in some patients undergoing total exenteration [24] and our study did not assess the postoperative quality of life where it has been shown that young age at exenteration contributes to disturbed body image and impaired sexual functioning but with acceptable emotional functioning [25]. **None of our patients were sexually active during the period of follow-up. It is well known that the more extensive the pelvic surgery for cancer, the greater the effect on body image and self-perception of attractiveness. The greatest restriction on postoperative quality of life appears to be imposed by such disturbances in sexual function, particularly where the surgery is non-reconstructive in nature **[26]. **In this respect, myocutaneous flap reconstruction appears in such patients to provide the greatest satisfaction with the lowest morbidity **[27]. **No one in our group underwent neovaginal reconstruction, where it has been suggested that sexual activity is able to be resumed in about half of the patients of whom over 80% will do so within the first postoperative year **[28]. In general, we would recommend the use of a pedicled TRAM flap with primary closure and selective neovaginal reconstruction in younger sexually active patients particularly when there is combined preoperative irradiation techniques or where intraoperative or repeat perineal irradiation is used.

## Authors' contributions

**Andrew P Zbar**: Assisted in the format and design of the paper and provided patients and surgical expertise

**Radhakanth K Shenoy**: Evaluation of the patients

**Antonio Chiappa**: Assisted in patient management and performed the literature search

**All authors read and approved the final manuscript**.
